# Prediction of host - pathogen protein interactions between *Mycobacterium tuberculosis* and *Homo sapiens* using sequence motifs

**DOI:** 10.1186/s12859-015-0535-y

**Published:** 2015-03-26

**Authors:** Tong Huo, Wei Liu, Yu Guo, Cheng Yang, Jianping Lin, Zihe Rao

**Affiliations:** State Key Laboratory of Medicinal Chemical Biology, Nankai University, Tianjin, 300071 China; College of Life Sciences, Nankai University, Tianjin, 300071 China; College of Pharmacy, Nankai University, Tianjin, 300071 China; Tianjin International Joint Academy of Biotechnology and Medicine, Tianjin, 300457 China

## Abstract

**Background:**

Emergence of multiple drug resistant strains of *M. tuberculosis* (MDR-TB) threatens to derail global efforts aimed at reigning in the pathogen. Co-infections of *M. tuberculosis* with HIV are difficult to treat. To counter these new challenges, it is essential to study the interactions between *M. tuberculosis* and the host to learn how these bacteria cause disease.

**Results:**

We report a systematic flow to predict the host pathogen interactions (HPIs) between *M. tuberculosis* and *Homo sapiens* based on sequence motifs. First, protein sequences were used as initial input for identifying the HPIs by ‘interolog’ method. HPIs were further filtered by prediction of domain-domain interactions (DDIs). Functional annotations of protein and publicly available experimental results were applied to filter the remaining HPIs. Using such a strategy, 118 pairs of HPIs were identified, which involve 43 proteins from *M. tuberculosis* and 48 proteins from *Homo sapiens*. A biological interaction network between *M. tuberculosis* and *Homo sapiens* was then constructed using the predicted inter- and intra-species interactions based on the 118 pairs of HPIs. Finally, a web accessible database named PATH (Protein interactions of *M. tuberculosis* and Human) was constructed to store these predicted interactions and proteins.

**Conclusions:**

This interaction network will facilitate the research on host-pathogen protein-protein interactions, and may throw light on how *M. tuberculosis* interacts with its host.

**Electronic supplementary material:**

The online version of this article (doi:10.1186/s12859-015-0535-y) contains supplementary material, which is available to authorized users.

## Background

Tuberculosis (TB), caused by *Mycobacterium tuberculosis* (MTB), is a major global health concern [1]. According to the World Health Organization (WHO) report [2], there were an estimated 8.7 million new cases of TB (13% co-infected with HIV) and 1.4 million TB-related deaths in 2011. Clearly, the number of TB-related deaths in single year is alarmingly higher than the roughly 300,000 deaths reported for the bird flu pandemic in 2009 [3]. Further, the regimens recommended for the treatment of TB are complex, often very long and include highly toxic drugs that have side effects. An antibiotic course consisting of four first-line drugs like isoniazid, rifampicin, ethambutol and pyrazinamide for six months is recommended for treatment of TB. These first-line drugs were discovered more than 50 years ago [2,4]. Drug discovery for TB continues to lag behind. Co-infection with retroviruses like HIV further complicates TB treatment. Emergence of multi-drug resistant and extensively-drug resistant strains of *Mycobacterium* has threatened to derail global efforts for reigning in this pathogen [5]. Therefore, there is an urgent need to develop new anti-mycobacterial drugs [4] through an understanding of the genetics and physiology of *M. tuberculosis*.

*M. tuberculosis* primarily infects the respiratory system where it encounters alveolar macrophages and dendritic cells patrolling the lungs. However, the bacterium has an uncanny ability to survive the onslaught and in fact it uses the host macrophages for replication [5]. Virulence factors like an unusual cell wall made up of mycolic acid, *UreC* gene that prevents acidification of phagosomes, and the ability of the pathogen to neutralize reactive nitrogen and oxygen intermediates using reductases helps the bacterium evade the host immune system. In addition to macrophages, T-cells have been shown to participate in host cell response against mycobacterium [6,7]. However, mycobacterium evades elimination by the host immune response and causes disease. Therefore, it is essential to study the interactions between *M. tuberculosis* and the host to learn how these bacteria cause disease [8]. The availability of the complete genome sequence of the pathogen *M. tuberculosis* [1] and the host *Homo sapiens* [9] provides an essential tool for prediction of these host-pathogen protein interactions.

Host-pathogen protein interactions (HPIs) are often involved in the pathogen’s strategy to invade the host organism, breach the host’s immune defenses, as well as replicate and persist within the organism [10,11]. Experimentally, there are two main approaches for detecting interacting proteins: binary approaches such as the yeast two-hybrid (Y2H) system and luminescence-based mammalian interactome mapping and co-complex methods such as co-immunoprecipitation (coIP) coupled with mass spectrometry (MS) [12]. However, these methods are time-consuming and expensive, especially when adopted in high-throughput mode [13]. Therefore, many computational methods have been developed to improve the coverage, accuracy, and efficiency in identifying protein pairs. These methods for predicting protein-protein interaction (PPI) take advantages of high-throughput data [14] and are based on protein sequence, structural and genomic features that are related to interactions and functional relationships [15,16], including phylogenetic profile [17,18], gene neighbor and gene cluster methods [19,20] and interologs [21,22]. Interologs, also referred to as homologous PPI method, is based on the assumption that homologous proteins preserve their ability to interact [23]. Recently, it has been applied for not only recognizing PPIs within an individual organism [24,25], but has also been used to detect host-pathogen protein interactions [26,27].

In this work, we developed a systematic flow to predict the HPIs between *M. tuberculosis* and *Homo sapiens* based on sequence motifs. First, protein sequences were used as initial input for identifying the HPIs between *M. tuberculosis* and *Homo sapiens* by ‘interolog’ method. The HPIs were further filtered by domain-domain interactions (DDIs) prediction. Then, protein functional annotations and existing experiments results were applied to remaining HPIs. As a result, 118 pairs of HPI were identified, which involve 43 proteins from *M. tuberculosis* and 48 proteins from *Homo sapiens*. Intra-species PPIs were further predicted for the proteins from *M. tuberculosis* and proteins from *Homo sapiens* using VisANT [28], Reactome [29], InteroPorc [30], IntAct [31], DIP [32], MPIDB [33], MINT [34], and HPRD [35]. A biological interaction network between *M. tuberculosis* and *Homo sapiens* was then constructed by the predicted inter- and intra-species interactions. Finally, a database named PATH (Protein interactions of *M.tuberculosis* and Human) was constructed to store these predicted interactions and proteins.

## Methods

### Identifying HPIs by sequence comparison

**Figure 1:**
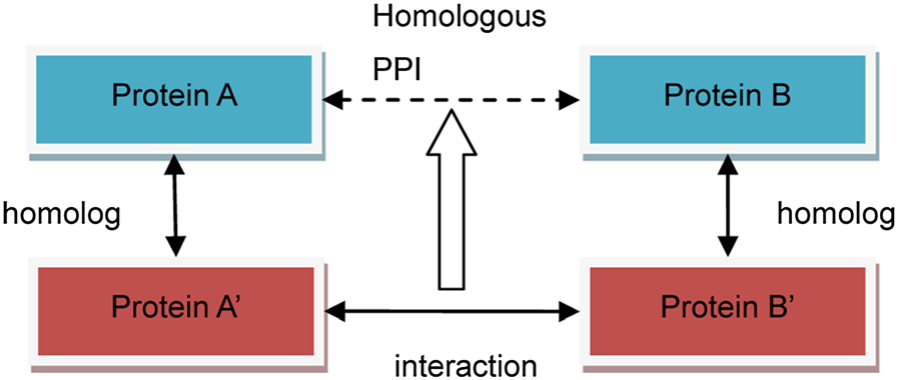
**Homologous PPI derived from interactions between homologs.** Protein A’ and B’ are the proteins which have direct interactions, while Protein A and B are their homologs, respectively. The interaction between A and B is called homologous protein-protein interaction.

Figure 1 shows the procedure used to identify HPIs. The procedure was based on the rationale underlying interolog [36], which implies that two proteins (A and B) are predicted to interact if their relative homologs (A’ and B’) interact.

To predict homologs, the basic local alignment search tool BLAST (Basic local alignment search tool) [37] was employed to compute sequence similarities. Query protein sequences were aligned against all sequences with known interactions stored in the databases BIPS [38] and HPIDB [39]. BIPS and HPIDB are integrated databases including several data sources such as DIP [32] and IntAct [31], and both of the databases allow the users to set the parameters freely. The e-value and identity parameters were set to 1e-10 and 30 respectively, and the source of target interactors was set to *Homo sapiens* (taxid:9606). The query protein sequences were obtained from TB database [40].

### Detecting domain-domain interactions (DDIs)

**Figure 2:**
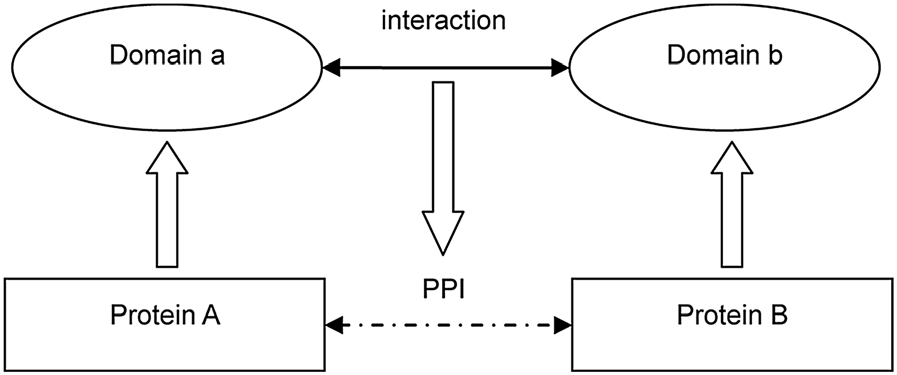
**Domain-domain interaction prediction.** Protein ‘A’ was predicted to interact with protein ‘B’ if A’s domain ‘a’ interacts with B’s domain ‘b’.

Domains play an important role in mediating protein-protein interactions [41,42]. The studies on DDI (domain-domain interaction) are based on the assumptions that: (1) DDIs are independent of each other, and (2) two proteins interact if at least one pair of domains from two proteins interacts. DDI were constructed in three steps - 1) the protein sequences of *Homo sapiens* and *Mycobacterium tuberculosis* were assigned to families or domains; 2) a whole domain-domain interaction network was drawn; 3) mapping the domain ‘a’ from protein ‘A’ in *Homo sapiens* and domain ‘b’ from protein ‘B’ in *Mycobacterium tuberculosis* to the whole network. If the domain ‘a’ interacted with domain ‘b’, the protein ‘A’ was predicted to interact with the protein ‘B’ (as in Figure 2).

To identify DDIs, the proteome of *M. tuberculosis* and *Homo sapiens* was aligned with Pfam families or domains with an E-value cut-off of 1e-10 using the Pfam-map program [43]. Then, protein-domain databases including 3DID [44], iPfam [45], DOMINE [46], DAPID [47] were selected to draw the DDI map.

### Filtering HPIs by biological context or functional annotation

The information of each protein in the HPI pairs (including subcellular location, tissue specificity, biological process, molecular function, and cellular component) was obtained from the Uniprot website (www.uniprot.org). If the functional annotation of the pair of interactors in the quasi-credible HPI was found to correspond with at least one of the defined terms, the quasi-credible HPI was selected and upgraded as credible HPIs. The terms were selected from previously published studies on the infection and pathology of MTB [48-51].

### Identifying intraspecific PPI network in *Homo sapiens* and *Mycobacterium tuberculosis*

The protein A from *Homo sapiens*, and protein B from *Mycobacterium tuberculosis*, that were involved in a HPI, were further screened against PPI databases to identify intraspecific PPIs. The resource of the intraspecific PPI for *Mycobacterium tuberculosis* included VisANT, Reactome, InteroPorc, IntAct, DIP, MPIDB, MINT, whereas for *Homo sapiens*, IntAct, HPRD, MINT, Reactome, DIP were included. We also attempted to use more databases such as virusmint [52], virhostnet [53], and STRING [54], while the number of PPIs would not be increased due to the overlaps and redundancy among the databases.

## Results and discussion

**Figure 3:**
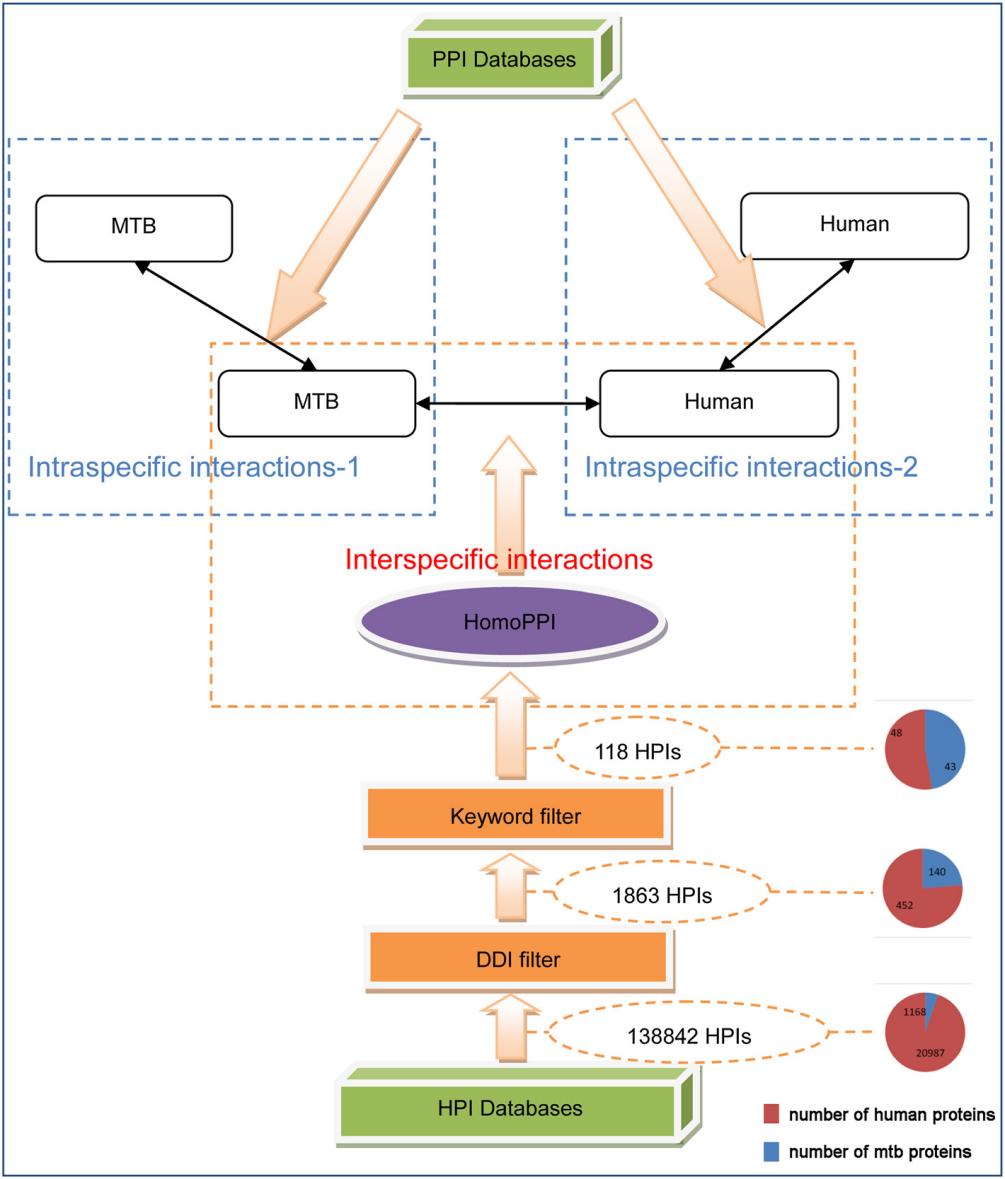
**The systematic flow of HPI prediction.** The Homologous HPI (HomoHPI) were obtained from the HPI databases (HPIDB, BIPS) by BLAST method, followed by applying the DDI filter and keyword filter. After applying these filters, the number of HPIs was trimmed from 138842 to 1863 and then to 118, respectively. The pie charts on the right depict the components of PPIs obtained from each procedure. The intraspecific interactions were extracted from 8 PPI databases (VisANT, Reactome, InteroPorc, IntAct, DIP, MPIDB, MINT, HPRD) and narrowed down to non-redundant data.

Figure 3 shows the schematic flow for predicting HPIs beginning with *Homo sapiens* and *Mycobacterium tuberculosis* protein sequences. 138842 pairs of HPIs were obtained after a BLAST search, then 1863 pairs of HPIs were obtained after DDI filtering, and finally 118 pairs of HPIs were identified after keyword filtering, which involved 43 TB proteins and 48 human proteins.

### The interspecific interactions between *Homo sapiens* and *Mycobacterium tuberculosis*

**Figure 4:**
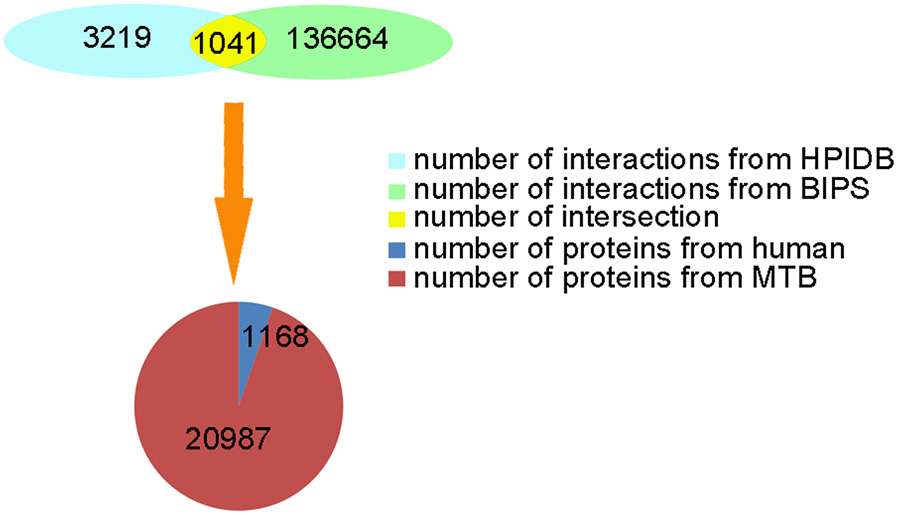
**Procurement of initial data.** The original HPI data were from HPIDB and BIPS database, and it included 1,168 MTB proteins and 20,987 human proteins.

The HPIs between *Homo sapiens* and *Mycobacterium tuberculosis* (MTB) were predicted based on sequence motifs, using HPIDB and BIPS. By performing a BLAST search of the two databases, 3219 HPIs were obtained between *Homo sapiens* and *Mycobacterium tuberculosis* from HPIDB, and 136664 HPIs from BIPS, with 1041 overlapping HPIs between the two databases. In total, there were 138842 non-redundant HPIs involving 1168 MTB proteins and 20987 human proteins (Figure 4).

Furthermore, the 138842 HPIs were filtered by applying the DDI filter. After aligning the 1168 MTB protein sequences and 20987 human protein sequences to domain or family, 3498 host-pathogen (human-MTB) specific DDIs were extracted (Table 1). Further, by removing redundant HPIs, 1863 non-redundant HPIs were obtained involving 140 MTB proteins and 452 human proteins (Additional file 1: Table S1).

**Table 1:** **The human-MTB specific DDIs in different databases**

**DDI databases**	**Number of Host-pathogen DDIs**
DOMINE	1928
DAPID	62
3DID	601
iPfam	907

Functional annotations of a protein are important and useful to understand the biological properties. Previous studies indicated that surface proteins consisting of secreted and membrane proteins could play a central role in the interaction of the pathogen with its environment, especially in the pathogenicity of MTB [55], and the term “membrane” was usually used to filter the functional annotation [56-58]. The immune system associated proteins of *Homo sapiens* would also contribute to the host-pathogen interactions [59]. Therefore, functional annotations and biological properties were used to further filter 1863 pairs of predicted HPIs. The “keyword filter” was applied to identify the functional annotation of proteins [60]. The keywords used were “membrane” for filtering *Mycobacterium tuberculosis* proteins, whereas “respiration”, “T cell”, “lymphocyte”, “phagocyte”, “lung”, “macrophage”, “dendritic cell”, “immune”, “B cell”, “alveol”, “toll-like receptor”, “bronchial epithelial cells” for filtering *Homo sapiens* proteins [48-51]. Each pair of HPI was retained only if both of its interactors corresponded to at least one of the above keywords. Finally, 118 pairs of HPIs were obtained by applying this filtering procedure involving 43 *Mycobacterium tuberculosis* proteins and 48 *Homo sapiens* proteins (Figure 3). All the proteins from *Mycobacterium tuberculosis* engaged in these 118 interactions were associated with the membrane, whereas among the 48 *Homo sapiens* interactors, 8 matched the keyword “T cell”, 5 matched by keyword “phagocyte”, etc (as in Table 2).

**Table 2:** **The “keywords filter” and its number of corresponding hits**

**Keywords**	**Number of corresponding proteins**
Membrane (MTB)	43
Respiration (human)	13
T cell (human)	8
Phagocyte (human)	5
Lung (human)	11
Macrophage (human)	3
Dendritic cell (human)	1
Immune (human)	11
B cell (human)	2
Toll-like receptor (human)	3

We checked the validity of these predictions by assessing the specificity and sensitivity. Random sets or true negatives were usually used for calculating the specificity [38,61]. In our work, we used the negatome database [62] as a source for non-interactions. 6532 non-interacting pairs from negatome as a reference set were processed by our method including sequence comparison and DDI detection. There were 618 pairs remained after the BLAST step, and they were further narrowed down to 376 pairs after DDI filter. Specificity was calculated as the percentage of correctly predicted true negatives out of 6532 non-interacting pairs. Thus the specificity of our method was 94.2% ((6532-376)/6532). Since gold-standard datasets of experimentally verified human-MTB PPIs are not readily available, we compared our predictions with previous reports to assess the sensitivity and accuracy. Our predictions included 23 MTB proteins (53.5%) that were suggested to play a significant role in the infection and intracellular survival [50,63-65]. In addition, we also enriched our results with the KEGG pathway and identified more proteins involved in the HPI such as Rv0934, Rv1411c and Rv3875 [66-68]. The coverage of our method depended on the previous experimental observations of similar interactions (template PPI), thus the coverage and accuracy would be increased as more template PPIs were identified.

To improve the accuracy, an increasing number of approaches have been developed taking advantage of the information residing in the motifs or structures. A structure-based interaction network between MTB and human was constructed recently emphasizing the importance of physical interactions [69]. This structure-based prediction could probably eliminate true negatives, while it was limited by the number of known protein complexes (templates). However, a simultaneous time-course microarray method was developed, which aimed at discovering the HPIs experimentally instead of solely depending on the known templates [70,71]. The experiment-based method would make biological sense, while the application of the microarray may not be easy and convenient to any species. All in all, each method would have a good performance in some aspects, and the credibility of known templates was the key point to the “interologs” predictions that mainly based on the sequence comparison.

### The intraspecific interactions among *Homo sapiens* and *Mycobacterium tuberculosis*

For the 43 proteins of *Mycobacterium tuberculosis* and 48 proteins of *Homo sapiens* in the host-pathogen interactions, intraspecific interactions were further studied. The interactions of *Mycobacterium tuberculosis* originated from 7 databases: VisANT, Reactome, InteroPorc, IntAct, DIP, MPIDB, and MINT, whereas the *Homo sapiens* interactions originated from 5 databases: IntAct, HPRD, MINT, Reactome, and DIP. By removing the redundancy from various data sources, there were 587 direct intraspecific interactions in *Mycobacterium tuberculosis* containing 374 MTB proteins and 7157 interactions in *Homo sapiens* containing 3062 human proteins.

### Host-pathogen interaction map and key proteins

**Figure 5:**
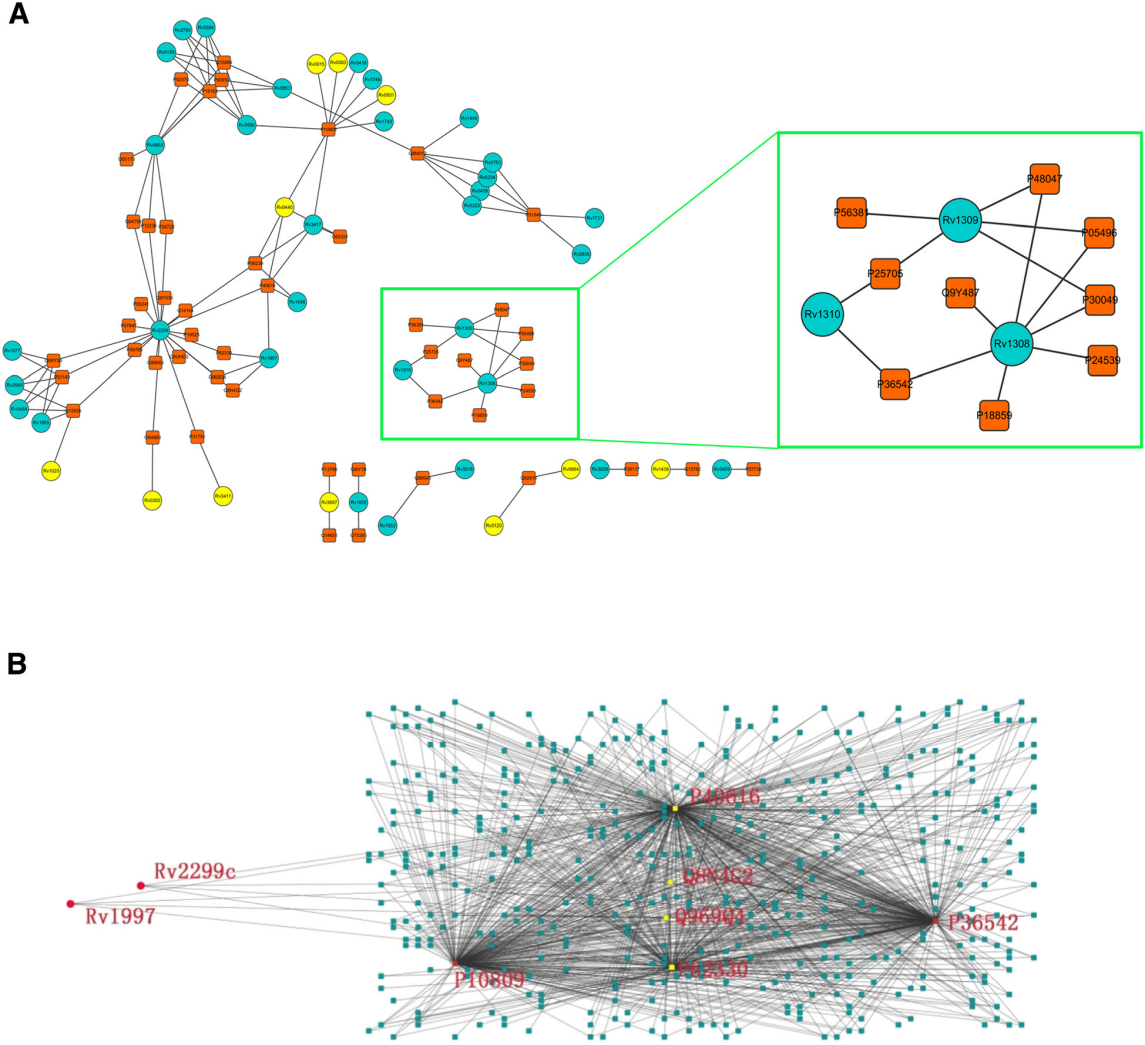
**Host-pathogen interaction map. A)** The cyan circles represent MTB proteins, while orange rectangles represent human proteins. The interactions are drawn as black lines, and the identified drug targets are colored yellow. An enlarged view of an interaction “island” (inset). **B)** A subnet involving rv1997, rv2299c, P40616, Q969Q4, P62330, and Q8N4G2. The map on the right was a human intraspecific interaction map. Points in yellow represent human proteins P40616, Q969Q4, P62330, and Q8N4G2. The other points are the direct interactors of these 4 proteins, and in these points, P10809 and P36532 as significant “hubs” in this sub-network are drawn in red points. Visualization was done with using Cytoscape [80].

By combining inter-specific interactions with intra-specfic interaction, a host-pathogen interaction map was constructed (Figure 5A). MTB proteins rv1308 (atpA), rv1309 (atpG), rv1310 (atpD), which were reported to play significant roles in MTB resistance [72], formed a small “island” in the interaction network by sharing common interactors (Figure 5A), which indicates that these proteins could cooperate with each other to interact with human proteins. MTB protein rv2299c (HtpG), which was predicted to have 20 potential interactors in the network, was previously reported to affect the dormant phase of *M. tuberculosis* [73]. Its interactors, such as P09769, Q14164 and Q9UHD2 protein in human, were identified to be involved in host immune responses based on the functional annotations, which indicated that rv2299c may engage the human immune system. MTB protein rv1997 (ctpF) was detected to be strongly induced during infection of human macrophages [74]. Four interactors (P40616, P62330, Q969Q4, Q8N4G2) of rv1997 (ctpF) mapped in the interaction network were either expressed in the lungs or were involved in immune responses based on the ontology annotation. Figure 5B shows a subset of the interaction map of proteins rv2299c and rv1997, which were also found to share 4 common interactors (P40616, Q969Q4, P62330, and Q8N4G2). These results indicate that proteins rv2299c and rv1997 are essential to understand how MTB survives the host immune response. In addition, human proteins P10809 and P36542 were considered as significant “hubs” and have more interactions in this sub-network. P10809 was previously identified as a key factor, which could influence B cell proliferation, T cell activation and macrophage activation [75-77]. Furthermore, 10 potential drug targets reported before [78,79] were also identified in our network (Figure 5A). It was noteworthy that 4 MTB targets shared the interactor P10809, which suggested that the human protein P10809 was critical in the MTB infection. Therefore, the predicted HPI map would throw light on how the MTB proteins affect the human cells.

### The structure of PATH

**Figure 6:**
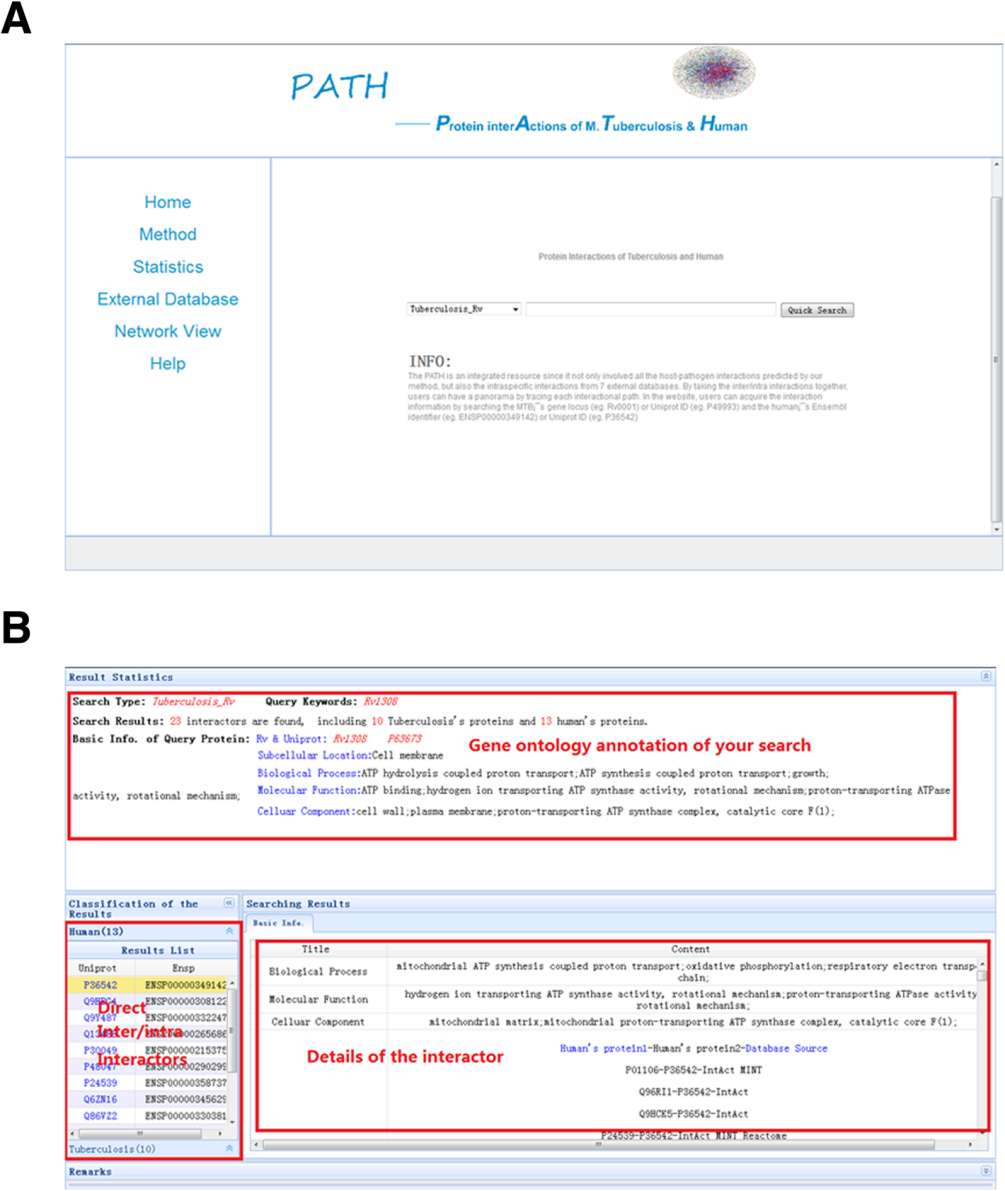
**Snapshot of the PATH website. A)** The homepage of the website. Users can acquire interactions information by searching the keywords. **B)** The information of the interactions. It also includes gene ontology annotations from MTB and humans.

Although there were many predictions focusing on the HPIs, only a few accessible databases were constructed. To store the predicted host-pathogen-interaction data, we developed a web-accessible database named PATH (Protein interactions of *M. tuberculosis* and human), which contains not only all the predicted host-pathogen interactions, but also the intraspecific interactions predicted from 7 external databases. Using the web-interface, users can acquire protein-specific interaction information by searching MTB’s gene locus (*eg*. Rv0001) or Uniprot ID (*eg*.P49993) and the human protein’s Ensembl identifier (*eg*.ENSP00000349142) or Uniprot ID (*eg.*P36542) (Figure 6A). The information of interactors both from interspecific network and intraspecific network can also be found during the keyword search (Figure 6B). In addition, the database will be enriched with new HPIs as soon as possible. PATH was built on an Nginx with Python and a MySQL Server as the back-end. HyperText Markup Language (HTML), JQuery and Cascading Style Sheets (CSS) were used at the front-end. It is freely accessible at http://cadd.pharmacy.nankai.edu.cn/tbdb. The web server and all parts of the database are hosted at College of Pharmacy, Nankai University, China.

## Conclusion

In this work, we present a specific and integrated database (PATH), which is publicly available and incorporates the predicted interspecific and intraspecific interactions between *Homo sapiens* and *Mycobacterium tuberculosis*. To our knowledge, PATH is the first specialized database for HPIs on *Mycobacterium tuberculosis*. Our interactions prediction model combined *in silico* algorithms with biological functional annotations. In this study, 118 credible HPIs were identified and stored in the PATH database. In PATH database, users can acquire the interspecific and intraspecific interactions between MTB and human and their related protein interactors by keyword search. The PATH database might facilitate understanding of mechanisms that causes TB, hence help to develop new therapeutic intervention tools for TB.

## Additional file

Additional file 1: Table S1. The PPIs derived from DDIs.
