# Supplementary material for: Prediction of host - pathogen protein interactions between Mycobacterium tuberculosis and Homo sapiens using sequence motifs
Source: BMC Bioinformatics. 2015 Mar 26;16(1):100. doi: 10.1186/s12859-015-0535-y (PMC4456996; doi:10.1186/s12859-015-0535-y)
Supplement: Additional file 1: Table S1. — The PPIs derived from DDIs. [file 12859_2015_535_MOESM1_ESM.docx]

Table S1. The PPIs derived from DDIs.

| PPI Intersection of DDI databases | | Number of protein-protein interactions |
| --- | --- | --- |
| Cross 4 databases | DOMINE&DAPID&3DID&iPfam | 31 |
| Cross 3 databases | DOMINE&DAPID&3DID | 0 |
|  | DOMINE&DAPID& iPfam | 1 |
|  | DOMINE&3DID&iPfam | 20 |
|  | DAPID&3DID&iPfam | 359 |
| Cross 2 databases | DOMINE&DAPID | 0 |
|  | DOMINE&3DID | 32 |
|  | DOMINE&iPfam | 23 |
|  | DAPID&3DID | 0 |
|  | DAPID& iPfam | 4 |
|  | 3DID&iPfam | 78 |
| From 1 database | DOMINE | 1260 |
|  | DAPID | 6 |
|  | 3DID | 22 |
|  | iPfam | 27 |
| Total number | | 1863 |
